# CRISPR-Cas9 Editing of Grapevine Clade V-Related *MLO* Genes: Grapevine Powdery Mildew Resistance (*Erysiphe necator*) and Plant Development Effects

**DOI:** 10.3390/plants15203168

**Published:** 2026-10-16

**Authors:** Satyanarayana Gouthu, Laurent G. Deluc

**Affiliations:** 1 Department of Horticulture, Oregon State University, Corvallis, OR 97331, USA; gouthus@oregonstate.edu; 2 Oregon Wine Research Institute, Oregon State University, Corvallis, OR 97331, USA

**Keywords:** CRISPR-Cas9, clade V *MLO* genes, grape powdery mildew resistance, chimerism, pleiotropic effects

## Abstract

Clade V-*MLO*s are associated with powdery mildew susceptibility in several plants. In grapevine, four *MLO* genes (*MLO3*, *MLO4*, *MLO13*, and *MLO17*) belong to clade V and have been studied to determine their contribution to susceptibility to *Erysiphe necator* (Grape Powdery Mildew—GPM). Using somatic embryogenesis to generate knockout mutants in grapevine, we performed a series of multiplex gene-editing-mediated transformations to determine each clade V-related *MLO*’s contribution to GPM resistance. We characterized a hierarchy of contributions from the four *MLO*s to GPM, with *MLO17* playing the largest role, followed by *MLO3* and *MLO13*. In addition, *MLO4* was a less essential modifier of GPM resistance: its loss-of-function, in combination with other *MLO*s, conferred a modest but consistent improvement in GPM resistance, and *mlo* mutants with a fraction of WT *MLO4* allele chimerism lacked the *mlo*-associated developmental pleiotropic effects. Defects in flower development in most *mlo4* mutant lines suggest a role for this gene in reproductive tissues. Our data also indicated functional redundancy within the clade V-*MLO*s, particularly between *MLO13* and *MLO17*. Ultimately, our results show that a certain degree of chimerism (wild-type background in the mutants) is necessary to achieve resistance to GPM without major growth defects or yield penalties. Ultimately, this approach offers a viable strategy for breeding powdery mildew-resistant grapevines while maintaining agronomic fitness.

## 1. Introduction

MLO (mildew Locus O) family proteins have been identified in several plant species, including 17 MLOs in grapevine [1], and some have been shown to affect infection by fungi that cause powdery mildew (PM) [2,3,4,5]. The MLO orthologs responsible for PM susceptibility are conserved across plant species and generally cluster in clades IV and V [1,6,7]. MLOs are required for pathogen–host cell penetration and negatively regulate plant defenses against fungal invasion. MLO proteins colocalize with and directly or indirectly influence the plasma membrane-localized soluble N-ethylmaleimide-sensitive factor attachment receptor (SNARE) penetration-resistance proteins (AtPEN1, AtPEN3, HvROR2, VvPEN1), which deposit cell wall reinforcements to act as physical barriers against fungal penetration [8,9,10]. Loss of function at the MLO locus confers resistance to the powdery mildew pathogen by blocking pathogen entry [10].

MLO-based resistance to powdery mildew is naturally occurring or can be introduced by silencing *MLO* genes in a broad range of plant species [2,11,12,13]. Beyond modulating PM resistance, MLO family proteins are involved in a wide range of physiological processes, including calcium signaling, root morphogenesis, and responses to biotic and abiotic stresses [14,15,16]. As a result, both natural and induced loss-of-function mutations in *MLO* genes produce pleiotropic effects, such as spontaneous necrotic leaf spotting, increased susceptibility to other diseases [17], and reduced yield, among others. Therefore, although *mlo*-based resistance is an attractive target for plant breeding programs, its use in agriculture is limited mainly by the yield penalty from growth defects associated with these mutations. However, two studies have demonstrated that the severity of growth defects can be reduced by partially restricting *MLO* expression and by residual *MLO* wild-type activity, the natural variant of *mlo11* in barley [18,19]. Likewise, a gene-edited chimeric *edr1* (Enhanced resistance1) mutant, another PM susceptibility gene in grapevine, expressing wild-type and mutant alleles, was reported to develop normally because of lower editing frequency in meristem tissues than in older tissues that display PM resistance [20].

In grapevine, researchers have investigated *MLO* genes involved in PM susceptibility based on their homology to functionally validated mildew susceptibility genes *AtMLOs 2*, *6*, and *12* [2], which are co-orthologs of the natural barley *mlo* allele [21]. In *Arabidopsis*, complete protection against PM requires silencing all clade V *MLO*s *(2*, *6*, and *12)* because of high functional redundancy. Early grapevine studies focused on identifying *MLO* genes involved in PM infection through induction kinetics [1,22], followed by loss-of-function or knockdown studies [4,5,23]. Complementation experiments with grapevine MLOs 3 and 4 partially rescued PM susceptibility phenotype in the PM-resistant *Arabidopsis* triple mutant, *Atmlo2*,*6*,*12*, and both were also shown to co-accumulate at the site of fungal penetration, along with PEN1 [9]. Grapevine RNAi experiments suggested a 77% reduction in PM severity associated with knockdown of *MLOs 3*, *13*, and *17* and no role for *MLO4* [4]. Using gene-editing approaches, the *VvMLO3* mutation has been reported to confer partial resistance to PM, accompanied by growth defects [5]. Near-complete resistance was reported with double mutations in *MLOs 13* and *17*, without pleiotropic effects, attributed to increased stilbene accumulation and improved oxidative scavenging [23].

In this study, we generated gene-edited lines carrying combinations of clade V *MLO* mutations to identify PM-susceptibility candidates and assess potential combinatorial effects on PM resistance. Based on resistance tests, mutant genotyping, and phenotype observations, the relationship between *MLO* gene combinations and resistance to powdery mildew, as well as the associated developmental defects caused by the loss of MLOs is partially elucidated. The results suggest that unintended chimerism, often associated with somatic embryogenesis following gene editing, might have alleviated the pleiotropic effects while retaining the benefits of *mlo*-based resistance in grapevine.

## 2. Results

### 2.1. Clade V mlo Genes in Grapevine and Induction During Powdery Mildew Infection

Using phylogenetic analysis, four grapevine MLOs were identified as the closest orthologs of functionally characterized PM-susceptibility MLOs from *Arabidopsis* and other species [1,22]. Two different gene annotations are currently used by the scientific community in the papers cited above (Table S1; [24]); we opted for Feechan’s annotation in this manuscript, i.e., *MLO 3*, *4*, *13*, and *17*. These four MLOs share the two conserved short C-terminal domains common to all MLOs, with confirmed roles in powdery mildew susceptibility in other dicot species [25,26,27,28]. Our microvine genome shows that *MLO13* and *MLO17* are adjacent, suggesting their paralogous nature (unpublished data) and their highly similar protein sequences.

**Figure 1:**
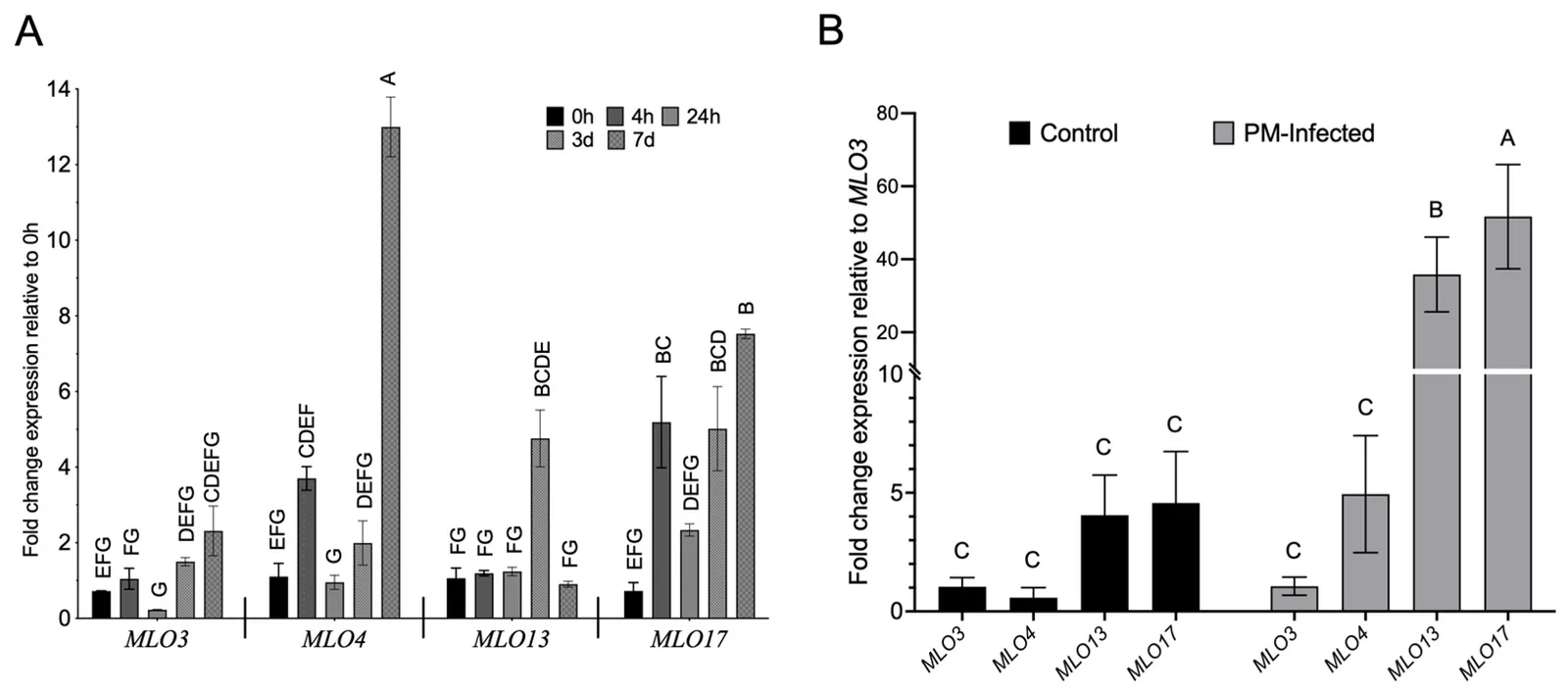
Real-Time PCR analysis of *VvMLO* gene expression in grape leaves in response to powdery mildew infection. (**A**). Expression fold changes were calculated relative to the 0 h sample for each *VvMLO* gene. (**B**). Expression fold changes in untreated control leaves (black bars) and infected leaves (grey bars) at 72 hpi relative to the *VvMLO3* baseline. Means were calculated from 3 to 7 biological replicates. Error bars represent the standard error of the mean (±SEM). For both panels, we performed a multi-factorial two-way ANOVA followed by global Tukey’s HSD post-hoc tests to evaluate differences among all groups. Different uppercase letters above the bars indicate statistically significant differences (*p* < 0.05) across the entire dataset within each respective panel. Treatment bars sharing at least one letter in common are not significantly different.

Transcript levels of the four candidate *MLO*s were quantified to determine their early- and late-stage responses to PM inoculation with established infection stages of *Erysiphe necator* (Table S2; [29,30,31]). The induction patterns of *VvMLOs 3*, *4*, *13*, and *17* in infected microvine leaves were observed from 4 h post-inoculation (hpi) to 7 days post-inoculation (dpi) (Figure 1A). While the early-stage transcriptional responses varied among the four *MLO* homologs, they were generally more highly expressed during colony expansion (3–7 dpi). During the 2–4 hpi period (conidial germination and attempted leaf penetration), *MLO4* and *MLO17* gene expression showed a significant ~4-to-5-fold increase (Figure 1A). At 24 hpi, when spores from haustoria and primary hyphae have formed, no significant changes were observed in the expression of the four *mlo* genes. At 3 dpi, when conidiophore development begins, only *MLO13* was highly expressed. At 7 dpi, *MLO4* and *MLO17* again showed significantly higher expression (Figure 1A). *MLO3* expression was largely unaffected by infection, or its induction was not captured during our sampling times. Given the steady-state pattern of *MLO3*, we used it to assess relative expression levels of *MLOs* in healthy and infected leaves at 3 dpi (Figure 1B). In non-infected leaves, *MLO4* expression was similar to that of *MLO3*, but *MLOs 13* and *17* were expressed at a 4-fold higher rate. The relative expression rate for *MLOs 13* and *17* was even higher in infected leaves, at 40- to 50-fold compared to *MLO3* (Figure 1B). Previous tissue-specific expression studies suggest that *MLOs 3* and *4* are predominantly expressed in floral and root tissues, indicating roles in developmental functions beyond PM interaction [1,22]; the pleiotropic effects we observed in the mutants, discussed later in the manuscript, support this notion.

### 2.2. CRISPR-Cas9 Editing Performance, Genotyping, Wild-Type Chimerism, and Pleiotropic Effects

PM susceptibility is attributed to the overlapping roles of clade V MLOs such as *AtMLO*s *2*, *6*, and *12* in *Arabidopsis* [2] and *SlMLO*s *1*, *5*, and *8* in tomato [28]. To assess each MLO’s contribution to PM susceptibility, we designed CRISPR-Cas9 constructs to generate single-, double-, triple-, and quadruple-knockout lines (Figure S1 and Table S3). In vitro cleavage assays confirmed the specificity of each sgRNA with no off-target cleavage among the other *MLO* homologs (Figure S2). We designed CRISPR constructs to generate all possible combinations of the *mlo* mutants. The failure to isolate six mutant combinations (*mlo4,13*, *mlo4,17*, *mlo3,4,13*, *mlo3,4,17*, *mlo4,13,17*) was due to poor performance of sgRNAs targeting MLO4 and MLO17 in multiplexed CRISPR constructs (Table S4). We incidentally retrieved double and triple mutants *mlo3,13* and *mlo3,13,17* while screening for quadruple knockout lines.

**Figure 2:**
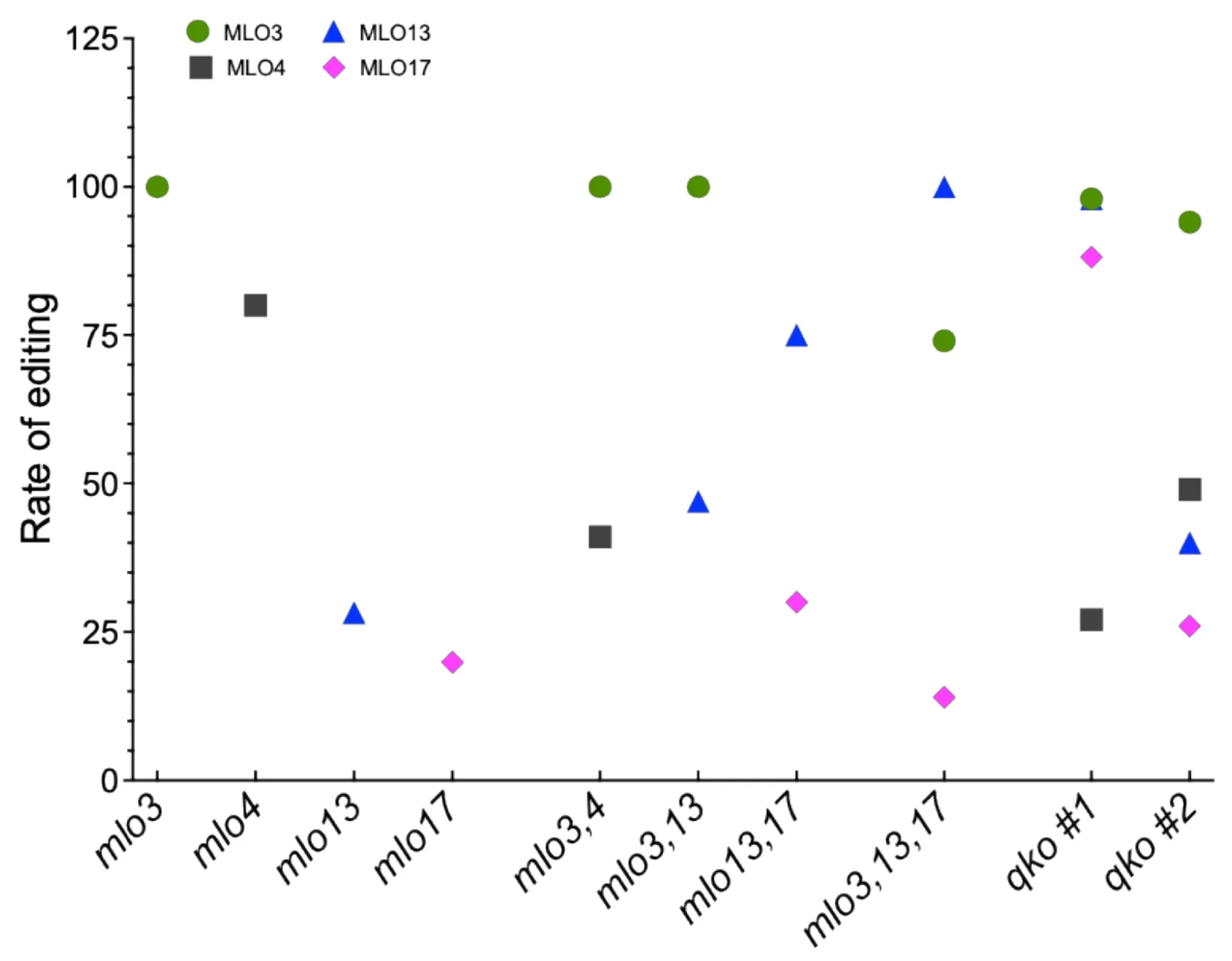
Gene editing rate in *mlo* mutants from DCODR analyses [32]. Cases where the editing rate is not either 100% (homozygous) or 50% (heterozygous) indicate chimerism of WT allele for that *MLO* gene.

We evaluated the transgenic lines for editing efficiency mainly using the deconvolution tool, DECODR [32], and defined their genotypes (Table 1). Amplicon sequencing of *mlo3*, *mlo4*, *mlo13*, *mlo17*, and *qko #1* lines was consistent with DECODR analyses within the 5% error margin (Figure S3) and confirmed the accuracy of editing rate, zygosity, chimerism, and mono- or biallelic nature derived from DECODR analyses. Bi- or monoallelic mutations were determined by the complete absence of the wild-type (WT) allele or ~50% contribution of the WT allele. We classified the mutation types as homozygous, heterozygous, or mosaic editing (more than two edited allele types) (Table 1 and Table S5). Mutants in which the WT *MLO* allele contribution was far from 50% (±10) mark are classified as chimeric plants. Overall, *mlo3* and *mlo4* were biallelic and completely edited lines, whereas *mlo13* and *mlo17* were chimeric, with genotypes showing ~75% of the reads related to the unedited WT *MLO* allele (Table 1). Among double mutants, *mlo13,17* was chimeric for *MLO17*. In *mlo3,4* and *mlo3,13* double mutants, the *MLO3* mutation was biallelic, whereas its counterparts were monoallelic. The quadruple mutant line, *qko #1* was chimeric with majority WT *MLO4* and very low WT *MLO17* allele and the editing in all four *MLOs* was of a mosaic nature (Figure 2 and Figure S5). *qko #2* was chimeric, with 75% wildtype *MLO17* with only 25% editing rate (Table 1, Figure 2). Incidental knockouts, *mlo3, 13* and *mlo3, 13, 17*, showed mono-, biallelic, and mosaic editing (Table 1, Figure 2).

In terms of pleiotropic effects, most mutants exhibited slow growth to severe growth defects, including arrested growth and non-flowering phenotypes, and some did not survive past tissue culture stages. Because dynamic traits such as early leaf senescence, transient stunting, and mutant survival cannot be fully captured by static evidence, we documented these chronological observations of developmental defects associated with each mutant and presented them in Table 1. *mlo13,17* showed major root growth defects and could not survive under greenhouse conditions (Table 1). *qko #2* was severely stunted, with premature leaf senescence in tissue culture, and did not survive the transfer to greenhouse conditions (Table 1). Only three mutant lines, *mlo3*, *mlo3,13*, and *qko #1*, lacked pleiotropic effects and showed growth, flowering, and fruit development identical to those of WT plants (Table 1). Despite the severity of developmental defects under soil conditions, all mutant lines listed in Table 1 could be regenerated and maintained in tissue culture. For this reason, to include all the mutants, we conducted disease resistance-related experiments using tissue culture-grown plants. After three cycles of growth, all the surviving mutants continued to grow in greenhouse soil conditions (Figure S6). However, *mlo4* and *mlo3,4* remained non-flowering, and *mlo17* maintained the slow growth phenotype. *mlo 3*, *mlo13*, *mlo3,13*, and *qko #1* show growth, flowering, and fruit production compared with WT plants (Figure S6).

### 2.3. Powdery Mildew Resistance in mlo Mutants

**Figure 3:**
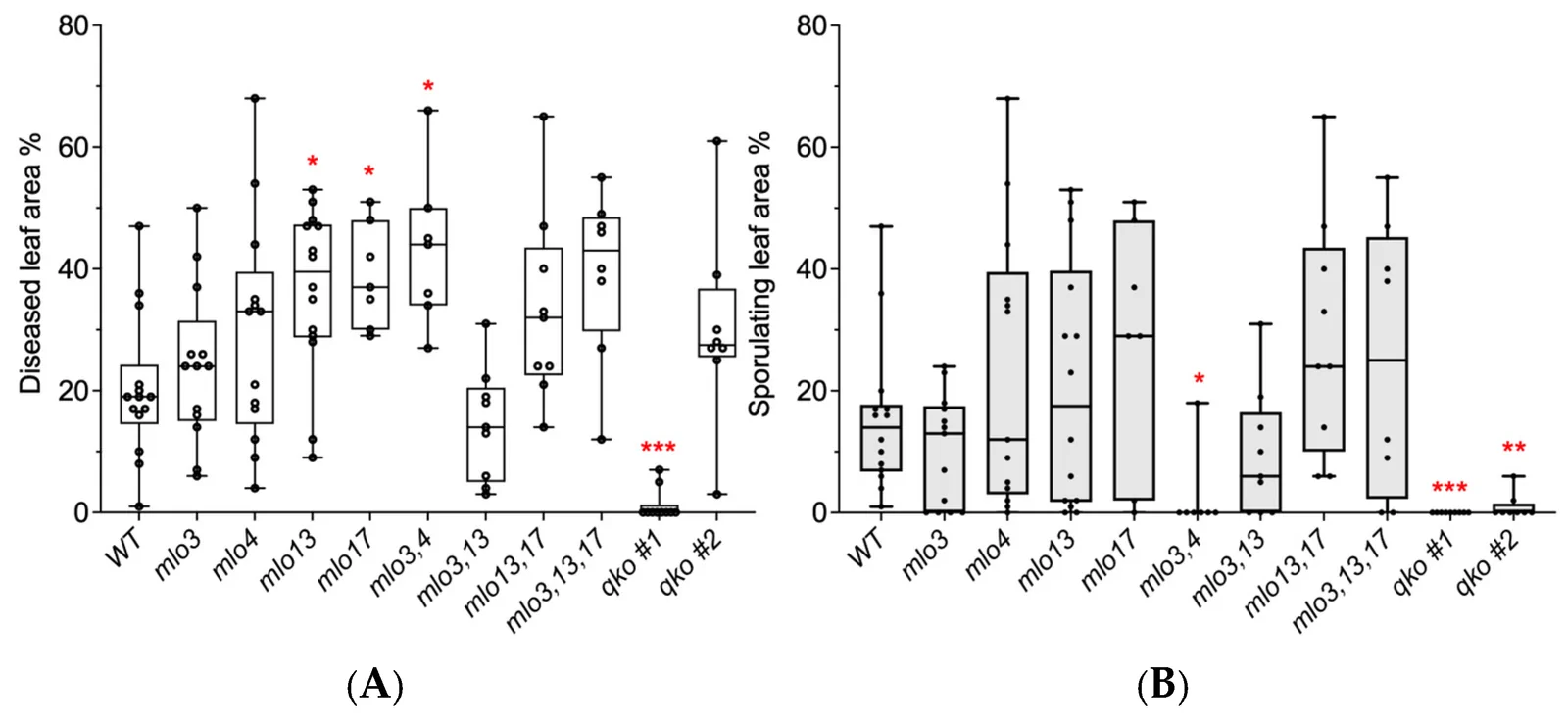
Quantification of the *E. necator* hyphal growth (**A**) and sporulation areas (**B**) in the *mlo* mutants. Leaf outlines, mycelial growth areas, and conidiophore growth areas were traced 72 h after inoculation with *E. necator* conidia using ImageJ software (version V1.54g) to determine total fungal growth area (cm^2^) and percentage sporulating area. Box plots display data from 10 to 14 infected leaves per genotype. Statistical significance was determined using two-tailed non-parametric Steel with Control tests for each dataset, comparing each *mlo* mutant group directly back to the WT control group. Asterisks indicate a statistically significant change compared to WT. Asterisks denote two-tailed significance levels (*: *p* < 0.05, **: *p* < 0.01, ***: *p* < 0.001) reflecting both significant increases and reductions relative to WT. In the box plots, the center horizontal line represents the median and the whiskers extend from the minimum to the maximum values, with all individual biological replicate data points displayed.

**Figure 4:**
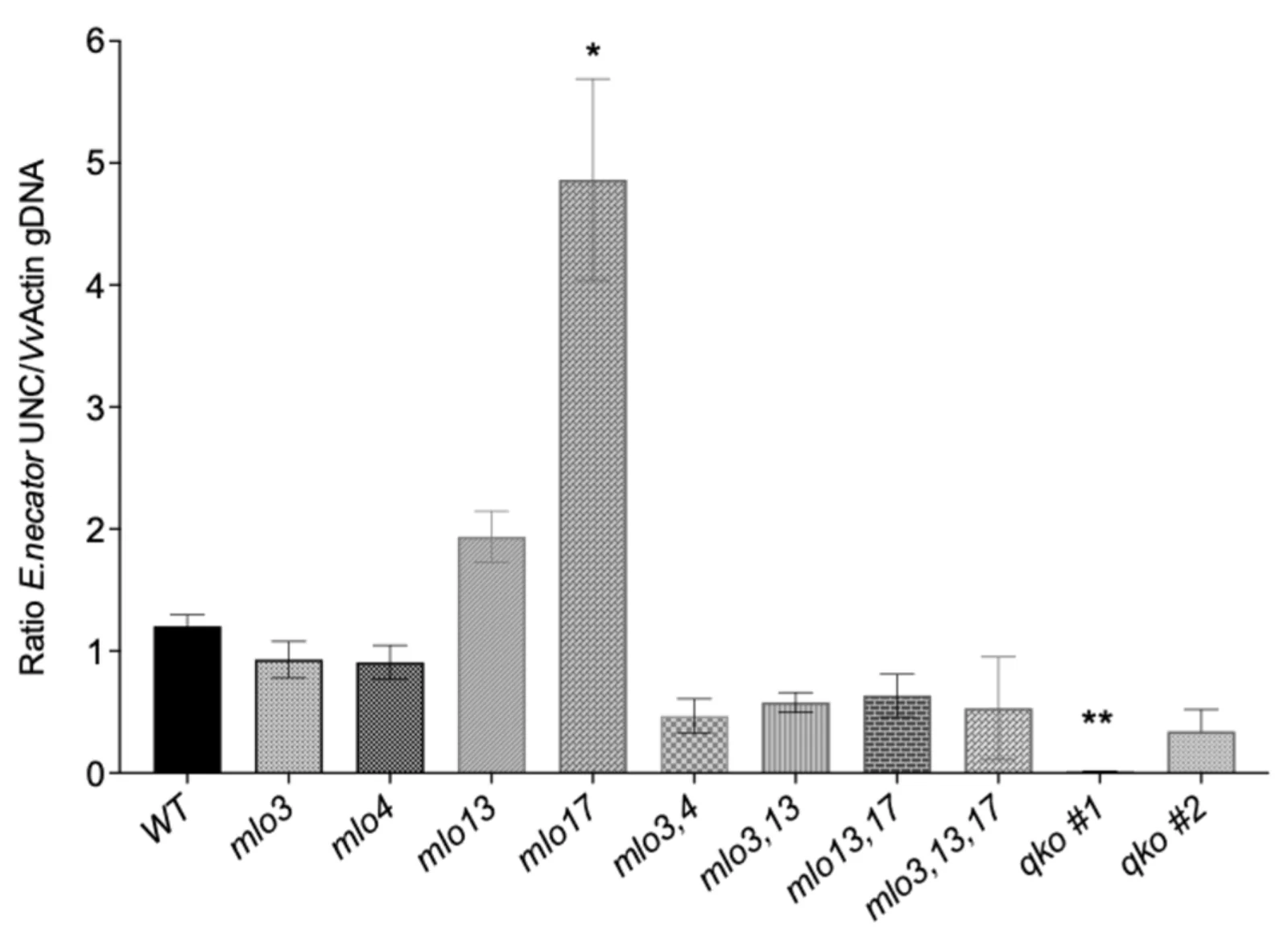
Evaluation of *E. necator* biomass in mutants two weeks post-inoculation (wpi). Ratios of *E. necator* to microvine gDNA in the leaves were determined quantitatively by qPCR with *E. necator* UNC gene and *Vitis* Actin gene primers using gDNA of infected leaves 2 wpi. Data represent the average of 7–12 leaves collected from 4 to 5 plants per experiment, across three independent inoculation experiments. Error bars represent ±SEM. Statistical significance was determined using a two-tailed non-parametric Steel with Control test, comparing each *mlo* mutant group directly back to the WT baseline group (black bar) to correct for simultaneous testing. Asterisks denote significance levels (*: *p* < 0.05, **: *p* < 0.01) reflecting both significant increases in fungal biomass (susceptibility) and significant reductions (resistance) relative to WT.

Resistance to PM was assessed based on mycelial growth and sporulation and fungal biomass in infected leaves (Figure 3, Figure 4 and Figure S5). Early microscopic observations of WT leaves inoculated with *E. necator* show that the majority of conidia had germinated and produced primary hyphae by 24 hpi (Figure S5). By 72 hpi, mycelial growth covered about 20–45% of leaf area, with 40–90% of it producing conidiophores (Figure 3). *mlo3* and *mlo4* showed similar levels of conidial germination and fungal growth, but sporulation in *mlo3* was at a reduced level compared to WT (Figure 3). In *mlo13* and *mlo17* mutants, hypervirulent hyphal growth was observed with significantly larger leaf area covered by powdery mildew than in WT (Figure 3). The rapid conidial germination and fungal growth in these mutants were evident, with secondary hyphae production at 24 hpi (Figure S5). *mlo13* and *mlo17* mutants are partially edited WT chimeric lines in which the majority of cells contain WT *MLO* alleles (Figure 2), and it is unclear how partially edited *MLOs* cause hypersensitivity to PM. Double mutation in *MLOs 3* and *4* increased hyphal spread area in *mlo3,4*, but mycelial growth was less dense, with significantly reduced sporulation (Figure 3). Mutation in *MLOs 3* and *13* resulted in decreased hyphal growth and unaffected sporulation in *mlo3,13* (Figure 3). By contrast, *mlo13,17* and *mlo3,13,17* showed increased mycelial spread in infected leaves compared to WT. The quadruple mutant line, *qko #2*, with partially edited *MLO17*, supported WT levels of fungal growth but significantly inhibited sporulation, similar to *mlo3,4*. Finally, the quadruple mutant line, *qko #1*, with completely edited *MLOs 3, 13*, and *17* and partially edited *MLO4*, showed complete inhibition of fungal growth (Figure 3). No conidial germination and emerging primary hyphae were seen in this mutant at 24 hpi (Figure S5). Out of multiple qualitative resistance trials with *qko #1*, only two isolated small-colony appearances were observed, confined to the leaf base and not spreading further. In conclusion, inhibition of fungal sporulation appears to be associated with mutations in *MLO3* and *MLO4*, but an additional *MLO17* mutation also reduces hyphal growth.

To quantitatively determine the fungal biomass supported by infected leaves at 14 dpi, we used the ratio of *E. necator* genome-derived 18S rRNA gene and the grapevine actin gene. Real-time PCR data showed no reduction in fungal biomass in any of the single mutants compared to WT leaves. Consistent with observed hypervirulent growth, *mlo13* and *mlo17* showed a significant increase in fungal biomass (Figure 4). The quadruple mutant line *qko #1*, with partially edited *MLO4*, supported negligible fungal biomass. These results indicate that achieving complete PM resistance may require mutations in all four MLOs in a specific ratio, with MLOs 3, 13, and 17 playing a significant role.

### 2.4. Expression Patterns of MLO Orthologs in the Mutants

**Figure 5:**
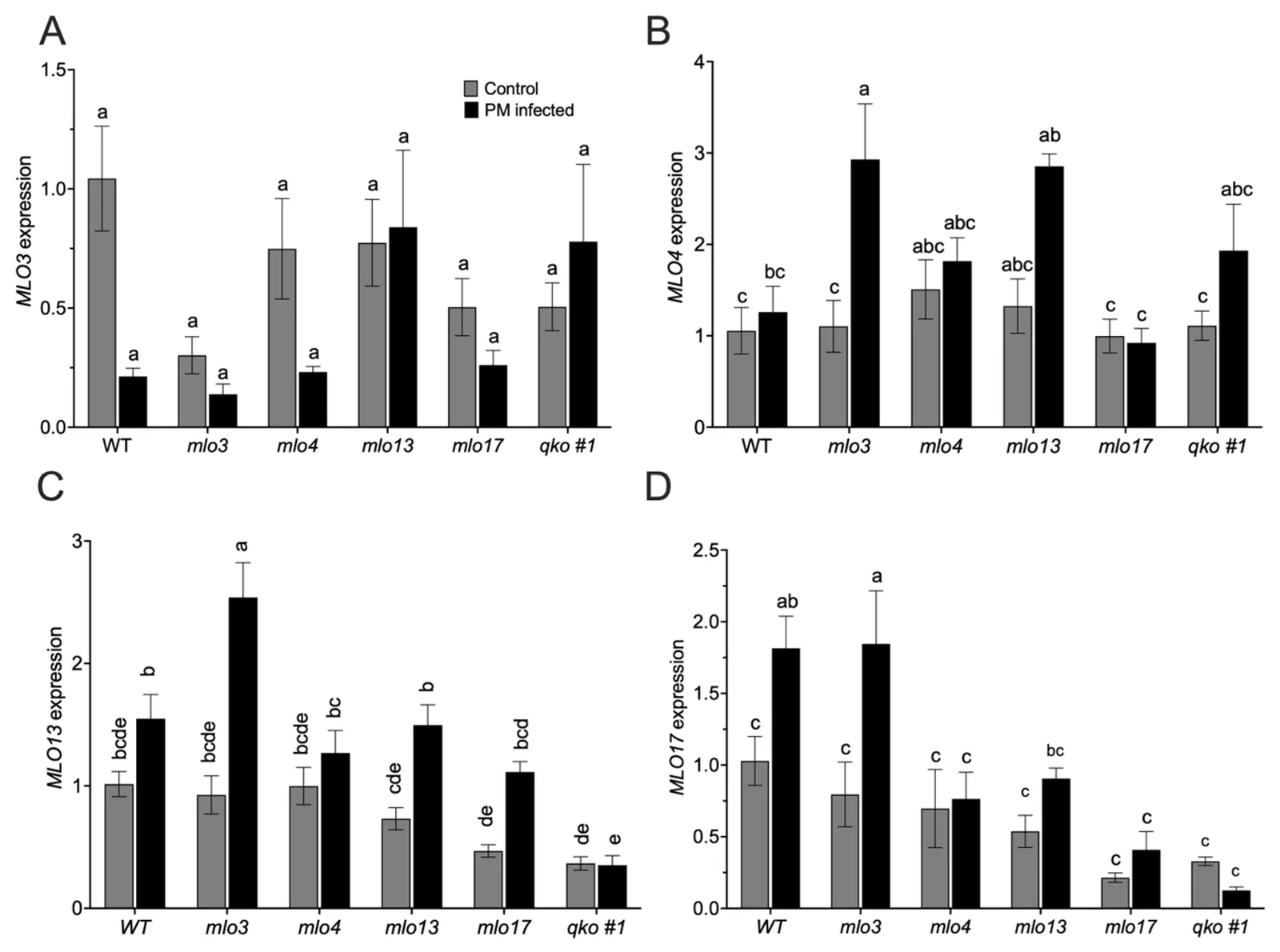
Early transcriptional response of *MLO* orthologs in non-infected and powdery mildew-infected WT and *mlo* mutants 72 hpi quantified through quantitative RT-PCR analyses. The results are expressed as fold changes relative to the expression in non-infected WT. To evaluate the multifactorial relationship between mutant and infection status, independent multifactorial two-way ANOVAs were performed for each gene, (**A**) *VvMLO3*, *p* = 0.1361, (**B**) *VvMLO4 p* = 0042, (**C**) *VvMLO13*, *p =* 0.0001, (**D**) *VvMLO17*, *p* = 0.0046, to evaluate the main effects of genotype, infection condition, and their interactive effects. Following the model evaluation, a comprehensive post-hoc Tukey’s HSD multi-comparison test was applied to each gene dataset independently to strictly correct for simultaneous testing across all treatment combinations. Statistically significant differences among all groups within each panel (*p* > 0.05) are indicated by a compact letter display above the bars. Treatment bars within the same panel that do not share any letters are significantly different from each other, comprehensively accounting for variation between genotypes and changes due to infection status. Means represent five biological replicates, and the error bars show SEM.

**Figure 6:**
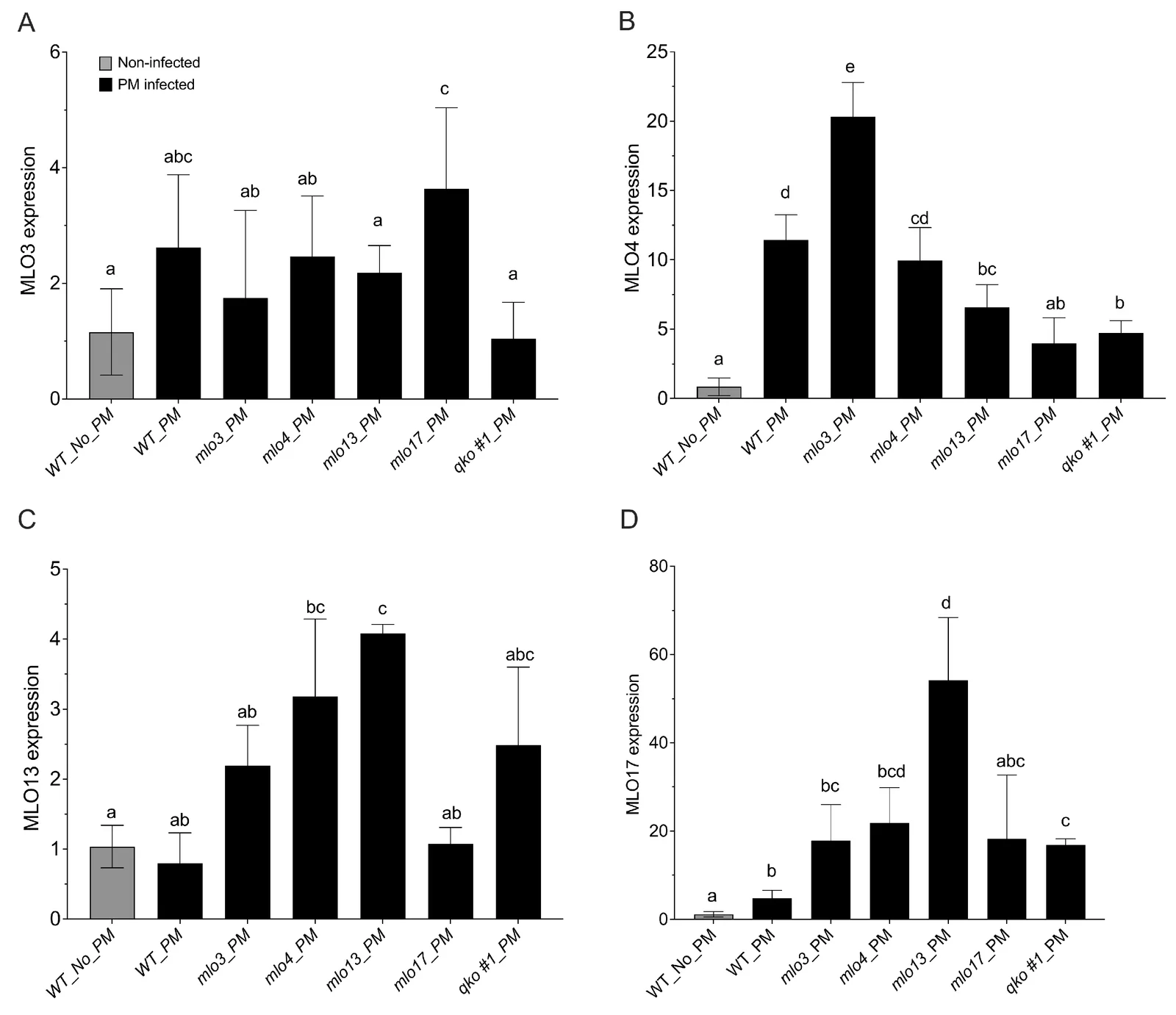
Late transcriptional response of MLO orthologs in WT and *mlo* mutants 2 wpi through quantitative RT-PCR analyses. The results are expressed as fold changes relative to expression in non-infected WT. (**A**), (**B**), (**C**), and (**D**) show the expression of *MLO3*, *4*, *13*, and *17*, respectively, in infected WT and mutants relative to noninfected WT. To assess statistical significance, model assumptions were evaluated using residual diagnostics. While most genes showed no substantial deviations, mild heteroscedasticity was detected for MLO13; therefore, the inference was based on a heteroscedasticity-robust standard error estimator. To correct for simultaneous testing across all treatment combinations and account for multi-factor interactions, a global post-hoc Tukey’s HSD analysis was performed. Means not connected by the same letter differ significantly (*p* < 0.05). Data represent means ± SEM (*n* = 5 biological replicates).

Transcriptional responses of the other *MLO* homologs in single knockouts and the *qko #1* mutant line were examined to determine any compensatory expression mechanism among the homologs as a result of known functional redundancy among MLOs during PM infection [2,33]. We examined early responses at 72 hpi in non-infected and infected mutants and WT (Figure 5) and long-term transcriptional responses were measured at 2 wpi (Figure 6). No significant expression changes in MLO genes were observed in mutant lines compared to WT in the absence of PM infection, indicating that the loss-of-function in one of the MLOs did not cause any compensatory response in other clade v homologs in the early transcriptional responses (Figure 5A–D grey bars). When challenged with PM, *MLO3* expression did not change significantly after loss of its homologs in *mlo4*, *mlo13*, and *mlo17* lines (Figure 5A). *MLO4* did not express in response to PM challenge in WT but was expressed at significantly higher levels in *mlo3* (Figure 5B). PM infection induced higher MLO13 expression in WT, but the increase was much more pronounced in *mlo3*, *mlo13*, and *mlo17* mutants (Figure 5C), suggesting that MLO13 might compensate for loss-of-function in MLOs 3 and 17. Under pathogen-challenged conditions, *MLO17* was induced in WT, but in all mutants except *mlo3*, *MLO17* expression was significantly lower (Figure 5D). While loss of MLO3, MLO13, and MLO17 in single mutants seemed to be compensated by one or more homologs, this was not apparent in the quadruple mutant, *qko #1*, except for substantially reduced *MLO13* and *MLO17* expression compared with WT under pathogen-challenged conditions.

The long-term expression response of *MLO3* at 2 wpi was not different from that of infected WT in any mutant line (Figure 6A). *MLO4* expression was maintained at a higher level in infected WT than in non-infected plants (~10FC), and similarly in infected *mlo3* and *mlo13*. However, *MLO4* expression was much higher in *mlo3* than in WT (Figure 6B). While early-stage induction of *MLO13* in *mlo3* and *mlo13* was maintained at the 2 wpi stage, it was also expressed at a higher level in PM-challenged *mlo4* at this stage (Figure 6C). The *MLO17* gene is by far the most highly induced in the mutants during the last stages of disease (Figure 6D). Consistent with its close genetic proximity to *MLO13*, significantly higher expression (50-fold higher) was observed in infected *mlo13* (Figure 6D). Taken together, these results indicate that *MLO4* may be expressed at a higher rate in response to the loss of MLOs 3 and 13 during early infection, and that this high expression is maintained through the mature stages of infection in *mlo3*. By contrast, *MLO13* is the most responsive gene to mutations in the other homologs at either the early or late stages of infection. MLO17 expression increased specifically in the chimeric mlo13 line due to the partial loss of its paralog.

## 3. Discussion

The induction of *VvMLOs 3*, *4*, *13*, and *17*, which represent the closest orthologs of *AtMLOs 2*, *6*, and *12*, followed previously reported trends in grapevine during PM infection [1,22] and the expression levels of *MLOs 13* and *17* much higher compared to *MLOs 3* and *4* at 72 hpi, indicating they might play major role in PM susceptibility (Figure 1B). PM-induced expression spanned the entire PM pathogenesis process (up to 1 wpi), and mutations in these MLOs affected key PM-infection stages, such as sporulation and hyphal growth. Among the transformants generated in our experiments, most genotyped mutants showed complex or mosaic editing patterns comprising several differentially edited cell lineages, as well as chimeric mutants with WT alleles (Table 1 and Figure S4). Mosaic editing and WT chimerism may arise from several events, including CRISPR plasmids introduced at or after the single-cell stage, delayed Cas9 activity, and random editing of cells at different stages in the proliferating embryogenic structure [34,35,36]. Nevertheless, differential editing rates of one or more MLOs enabled us to understand the roles of different MLO combinations in PM susceptibility, *MLO* resistance, and *mlo*-associated pleiotropic effects.

In the chimeric mutants *mlo13* and *mlo17*, which had a 25% target-editing rate (where 25% of the cells were mutated and 75% retained the functional wild-type allele), the presence of these remaining functional MLO alleles unexpectedly supported hypervirulent pathogen growth (Figure 3). We hypothesize that because *mlo*-mediated resistance is cell-autonomous, the interspersed arrangement of edited (resistant) and unedited (susceptible) cells within the leaf forced the fungal hyphae to forage across a wider surface area. This likely increased overall mycelial spread without a corresponding increase in sporulation. Partial loss of MLO function may also increase expression of a close paralog, making the plant more susceptible. By contrast, complete editing of *MLO17*, together with *MLO13*, in *qko #1* led to restricted mycelial growth, implicating MLOs 13 and 17 in promoting mycelial growth. (Figure 2 and Figure 3). Our data also suggest a combined role for MLOs 3 and 4 in fungal sporulation, as *mlo3,4* and other lines containing this combination showed significantly reduced sporulation (Figure 3). While MLOs were thought to be mainly involved in host cell penetration and fungal establishment, *mlo* mutations in *Arabidopsis* and tomato have been reported to reduce hyphal growth or conidiophore production, indicating a post-penetration role for MLOs during fungal development [2,11].

Previous studies in grapevine that generated non-chimeric mutants through gene editing reported partial to significant resistance to PM with single and double mutations in *MLO3* and *MLO13* [5,23]. In contrast, the single *mlo* mutants generated in our study showed no PM resistance, including the fully edited and non-chimeric *mlo3*, *mlo4*, and *mlo3,13* lines (Figure 4). Other double KO lines displayed partial resistance, such as reduced hyphal growth or sporulation, but lacked significant overall resistance to PM infection (Figure 3 and Figure 4). Complete PM resistance was achieved only when mutations occurred in all four MLOs, as seen in qko *#1* line. In contrast, the *qko #2* line showed only partial resistance, characterized by normal mycelial growth but inhibited sporulation and severe developmental defects (Figure 3). The nature of editing in *qko #1* is complex and mosaic: while *MLO 3* and *MLO17* are edited and completely non-functional in all the cells, the majority of cells carry an edited, non-functional *MLO17* (with only 12% of cells with a functional WT allele), where a large majority of cells (80%) retain the functional WT *MLO4* allele (Figure 2 and Figure S3). Despite its complete PM resistance, the *qko #1* line was entirely devoid of *mlo*-associated pleiotropic defects often seen in null mutations. The *qko #2* line differs from *qko #1* by having completely edited (non-functional) *MLO3* and *MLO4* genes combined with very low levels of editing in *MLO13* and *MLO17*—meaning the majority of its cells retain functional, PM-susceptible *MLO13* and *MLO17* (Figure 2). Comparing these *qko* genotypes and their distinct editing patterns strongly indicates that the *mlo17* allele contributes most to resistance, followed by the *mlo3* and *mlo13* alleles. This is consistent with the unequal genetic redundancy of *MLO*s reported in *Arabidopsis* with respect to PM resistance and developmental defects [2,33]. Similar *mlo*-based resistance with alleviated pleiotropic effects has also been reported in wheat due to a weaker mutation in a homolog [3]. Furthermore, PM resistance paired with reduced pleiotropic effects was previously associated with mosaic editing and WT chimerism in grapevine *edr1* mutants, whereas pure homozygous and bi-allelic *edr1* lines suffer from developmental defects [20].

Generating grapevine powdery mildew (PM)-resistant lines through complete null *mlo* mutations frequently carries high agronomic costs, as evidenced by the growth and developmental abnormalities observed in most of our mutants (Table 1). Common pleiotropic effects, including spontaneous necrotic lesions, accelerated leaf senescence, and stunted growth, align with the established roles of *MLO* genes in suppressing cell death pathways and delaying senescence, as well as their function as negative regulators of PM susceptibility [37]. Crucially, our results suggest that partial *MLO* activity resulting from mosaic editing and chimerism may provide a form of functional rescue, ensuring plant viability while maintaining complete PM resistance. This phenomenon is strongly supported by recent evidence in grapevine *edr1*mutants, where mosaic editing and wild-type chimerism conferred PM resistance without a growth penalty, whereas homozygous and bi-allelic *edr1* lines suffered severe developmental defects [20]. Similarly, the alleviated pleiotropic effects observed in natural barley *mlo-11* resistance and engineered wheat mutants have been attributed to residual wild-type *MLO* activity [3,18,19]. Taken together, these findings indicate that avoiding undesirable developmental penalties while maintaining robust resistance does not demand a strict ratio of chimerism; rather, it simply requires a threshold of temporal residual wild-type activity, such as MLO4 in this case, that controls essential developmental processes beyond PM susceptibility. Because of the complex and chimeric nature of our gene-edited lines, coupled with likely functional overlap among *MLO* homologs, accurately attributing specific pleiotropic defects to single *MLO* genes or combinations remains challenging (Table 1). Furthermore, the phenotypes are known to be heavily influenced by genotype-by-environment interactions [17,38,39]. Nevertheless, our phenotypic data provide important clues into MLO gene functions. For instance, *mlo4* and *mlo3,4* mutants with completely edited *MLO4* genes display non-flowering phenotypes (Figure S6). This suggests a predominant role for *MLO4* in vegetative and reproductive development that extends far beyond its accessory role in PM susceptibility. Conversely, lines with unedited or incompletely edited *MLO4* alleles were entirely free of flowering defects (Figure 2 and Table 1). This confirms that background activity of native MLO4 protein can prevent severe developmental penalties while simultaneously permitting enhanced disease resistance, as observed in the *qko #1* line.

Our study supports functional redundancy among clade-V *MLO*s, which explains why single and double mutants fail to exhibit PM resistance and why partial edits in some homologs prevent pleiotropic effects. The compensatory influence of one homolog or the other might partially rescue PM susceptibility or alleviate pleiotropic effects when *MLO*s with overlapping developmental functions are lost. Furthermore, functional redundancy among MLOs can span phylogenetic clades; for instance, in *Arabidopsis*, *AtMLO2* (clade V) and *AtMLO7* (clade III) can each partially compensate for the loss of the other’s function in PM susceptibility and pollen tube growth, respectively [40]. At the molecular level, unequal redundancy often results from active compensation mechanisms between homologs [41]. In the context of PM susceptibility, our data suggest that *MLO4* and *MLO13* compensate for the loss of *MLO3* (Figure 6B,C). Additionally, *MLO13* and *MLO17* appear to exert a long-term compensatory influence for the loss of other homologs (Figure 6C,D). Screening other MLO-related genes in these PM-challenged mutants could reveal the involvement of MLO genes outside clade V. Ultimately, this shows that maintaining a critical threshold of native MLO expression can decouple complete powdery mildew resistance from severe developmental penalties. This strategy provides a viable pathway for breeding disease-resistant grapevines without sacrificing agronomic fitness.

## 4. Materials and Methods

### 4.1. Microvine Plant Material and Embryogenic Callus

The microvine 04C023V0004 genotype used in this study, progeny from a cross between ‘Grenache’ × L1 ‘Pinot Meunier’ GAI1 mutant, described elsewhere [42], was provided by Dr. Mark Thomas (CSIRO Laboratories, Australia). The microvine plants were grown in greenhouses in a soilless medium with vermiculite under 16-h day and 8-h night conditions provided by supplemental lighting (100 μmol m^−2^ s^−1^, equivalent to 5400 lux) and slow-release granular fertilizer pellets. Initiation of embryogenic callus from immature anthers, maintenance media, and culture conditions were performed as described previously [43].

### 4.2. Guide RNAs and Design of CRISPR-Cas9 Constructs

CRISPR-Cas9 constructs were designed to generate single knockouts (*mlo 3*, *mlo 4*, *mlo 13*, and *mlo 17*), double knockouts (*mlo3,4* and *mlo13,17*), and a quadruple knockout (*mlo3,4,13,17*) using the CRISTA algorithm to rank sgRNA [44]. The last CRISPR construct was also intended to generate incidental double (*mlo4,13* and *mlo4,17*) and triple mutant combinations (*mlo3,4,13*; *mlo3,4,17*; *mlo4,13,17*; *mlo3,13,17*). Unique single guide RNAs (sgRNA) for single knockout mutants of *MLO*s 3, 4, 13, and 17 were selected from the 2nd or 3rd exon regions. A common sgRNA was designed for *MLO13* and *MLO17* for the double knockout *mlo13,17*, whereas individual sgRNAs of *MLO3* and *MLO4* were cloned into the CRISPR vector for the *mlo3,4* double mutant. A vector for the *mlo3,4,13,17* quadruple mutant (*qko*) was designed by multiplex cloning the sgRNAs of MLOs 3 and 4, and the common sgRNA of MLOs 13 and 17 into the plasmid (Figure S1). We used the single-transcriptional-unit (STU) CRISPR/Cas9 vector, pGEL031 (Addgene Plasmid #137900), with the eGFP gene to screen for transformed embryos. In the STU vector, Cas9 and sgRNAs are expressed from the maize ubiquitin Pol II promoter, and multiple sgRNAs are spaced with tRNAs to separate the individual sgRNAs post-transcription [45].

For single knockouts, we synthesized sgRNAs as duplex oligonucleotides, annealed them, and cloned them into BsaI-linearized pGEL031. To create double knockouts, the sgRNA scaffold was amplified by PCR using a pair of specific primers including two sgRNA sequences (BsaI-sgRNA1-F and BsaI-sgRNA2-R), and the purified PCR product was cloned. To express three sgRNAs for quadruple knockout, a two-sgRNA fragment and a third common sgRNA, generated by PCR amplification, along with the tRNA spacer sequence, were cloned together into pGEL031 via Gibson assembly.

### 4.3. Microvine Transformation

*Agrobacterium*-mediated transformation was performed following established protocols for the microvine embryogenic callus [43]. Briefly, embryogenic calli of microvine 04C023V0004 were co-incubated with *Agrobacterium tumefacien*s EH105 harboring the pGEL031 vector containing each sgRNA or combinations of sgRNAs (Table S3), and the infected calli were maintained on embryo-inducing medium with hygromycin selection. Six to eight weeks after the transformation, the emerging somatic embryos were screened for GFP fluorescence, and GFP-positive embryos were regenerated. Regenerated transformants were propagated and maintained in magenta boxes for genotype confirmation and further analyses.

### 4.4. Genotyping and Mutation Analysis

For mutant genotyping, the genomic DNA was extracted from the leaf tissue of regenerated plants using DNeasy Plant Mini kit (Qiagen, Hilden, Germany). PCR reactions were prepared using 50 ng genomic DNA as the template and specific primers designed to amplify a 200–300 bp amplicon spanning the expected double-strand break site of the target MLO gene. PCR products were purified using the Qiagen PCR purification kit (Qiagen) and sequenced using a nested primer (Table S6). Online analysis of mutant genotypes was performed by submitting the ab1 files from Sanger sequencing to the DECODR deconvolution tool to detect indels [32]. We sequenced the PCR amplicons by Oxford Nanopore long-read sequencing at Plasmidsaurus (Eugene, OR, USA). Approximately 5000 reads per sample were obtained from NGS and were analyzed using the CRISPR edits analysis tool (Geneious Prime, 2023.2.1).

### 4.5. Powdery Mildew Strain and Leaf Inoculation

*Erysiphe necator*, isolate E1-101, was maintained on detached leaves of Cabernet Sauvignon. To inoculate microvine leaves for PM resistance studies, we used conidia from 8-day-old colonies. Leaves of tissue-culture-grown microvine plants were transferred to water agar medium in Petri dishes, with their petioles inserted into the medium and a piece of wax paper placed over the leaves to prevent direct contact with the agar. For the induction experiment, conidia from a heavily sporulating source leaf were loaded onto a size 5/0 paintbrush and deposited on the entire leaf surface to achieve a uniform, high-density distribution and incubated at 25 °C until the specified collection times. The approximate saturation of the leaf area coverage was visually confirmed under a stereo microscope during inoculation.

### 4.6. MLO Induction Time-Course Studies

To test for induction of MLO genes upon PM inoculation, the leaves were uniformly coated with conidia using a paintbrush. RNA was extracted from the inoculated leaves, and cDNA was synthesized and used as a template for real-time PCR. Relative expression of MLOs was calculated using the ΔΔCt method [46].

### 4.7. Mycelial Growth and Sporulation Area Analysis

For the resistance studies, conidia were deposited on each side of the midvein and between the first and second lateral veins of a detached leaf (as above). Stereoscopic observations of initial spore germination and hyphal emergence were done at 24 hpi. We scored mycelial growth and sporulation at 72 hpi. Traces of the leaf area, the total area of the leaf with mycelial spread, and the area under sporulation were made on the lids of Petri dishes. The images were uploaded to ImageJ, and the fungal growth (cm^2^) was calculated as a percentage of total leaf area [47].

### 4.8. Real-Time PCR for Quantitative Fungal Biomass Analysis

Assessment of fungal biomass on infected leaves was performed 2 weeks post-inoculation (wpi) in a separate experiment using leaves spot-inoculated with *E. necator* conidia as described above. Quantitative assessment of fungal biomass in infected leaves was performed using real-time PCR to determine the ratio of fungal to microvine genomic DNA [48]. We extracted genomic DNA from infected leaves using the DNeasy Plant Mini Kit (Qiagen) and used it as a template for real-time PCR with primers for the *E. necator* 18S ribosomal RNA gene (GenBank: LC777882) and the *Vitis* actin gene. For qPCR, we prepared 20 μL reactions using the SsoAdvanced Universal SYBR Green Supermix Kit (Bio-Rad, Hercules, CA, USA) according to the manufacturer’s protocol. The amplification of the *E. necator*-derived amplicon was normalized to the microvine actin gene amplicon to quantify fungal biomass. The *E. necator*-to-microvine genomic DNA in mutants was expressed relative to the WT ratio.

### 4.9. Real-Time PCR for MLO Homolog Expression

To test for compensatory expression of MLO homologs in mutants, the leaves were spot-inoculated with *E. necator* conidia, and the inoculated leaves were collected at 72 h and 2 wk post-inoculation at specified times. We extracted RNA from the samples and synthesized cDNA for use as a template in real-time PCR. Relative expression of MLOs was calculated using the ΔΔCt method [46]. The primers used in real-time PCR are listed in Table S6.

### 4.10. Statistical Analyses

Data were analyzed using multi-factorial and single-factor linear models or non-parametric procedures depending on data distribution and experimental setup. For parametric gene expression analyses (Figure 1 and Figure 5), relative expression fold-changes were evaluated using standard multifactor least-squares linear models with fixed main effects and their corresponding interaction terms (Gene × Time for Figure 1; Mutant × Infection Condition for Figure 5). The significance of interaction terms was established using Type-III Analysis of Variance (ANOVA). Post-hoc simultaneous testing corrections across treatments within each dataset were performed using global Tukey’s HSD multi-comparison test (α = 0.05). Data represent means ± SEM from three to seven biological replicates. For late-stage gene redundancy expression analyses (Figure 6), data were modeled with mutant genotype as a main fixed effect and batch date as a blocking factor. To account for non-constant variance (heteroscedasticity) detected via residual diagnostics, model inference was built using heteroskedasticity-consistent covariance estimators (HC3). Simultaneous multi-group testing adjustments were conducted via Tukey’s HSD post-hoc test utilizing robust standard errors, with significant homogeneous groups summarized via a Compact Letter Display (CLD).

For non-parametric phenotypic and biomass quantifications (Figure 3 and Figure 4), data distributions did not satisfy normality or homoscedasticity assumptions. Consequently, simultaneous testing corrections against the Wild Type (WT) control group were executed using a two-tailed Steel with Control procedure (a non-parametric alternative to Dunnett’s test, α = 0.05), which also handles unequal sample sizes used in these experiments rigorously. Linear modeling and parameter estimations were conducted using the car R package (version 3.1.5), while simultaneous testing corrections, post-hoc adjustments, and compact letter displays were implemented using the emmeans and multcomp R packages.

## 5. Conclusions

Altogether, mutations in grapevine *MLO*s *3, 4, 13*, and *17* contribute unequally to PM resistance. Achieving PM resistance without severe detrimental effects requires a balanced mutant genotype that maintains a critical expression threshold from one or more partially functional homologs controlling other developmental processes. The editing profile of the resistant quadruple mutant *qko #1*, shows that the contribution of the *MLO17* gene to PM susceptibility is greater than that of *MLO3* and *MLO13*. Ultimately, our characterization of these mosaic lines demonstrates that complete PM resistance can be decoupled from severe developmental penalties. While the chimerism observed in our study occurred spontaneously during tissue culture, these lines provide a proof of concept for balancing immunity and plant growth without compromising agronomic fitness or yield. Future research should evaluate the chimeric lines under field conditions and assess durability across different environmental contexts.

## Figures and Tables

**Table 1:** Mutant genotypes, zygosity, and chimerism with observational pleiotropic effects in mutants through the developmental period.

Mutant	Target Gene	Type of Editing	Mutation Type	Plant	Phenotype in Soil Conditions
*mlo3*	3	Bi	Homozygous		
*mlo4*	4	Bi	Homozygous		Slow growth, non-flowering.
*mlo13*	13	-	Mosaic	Chimeric with WT	Severe leaf necrotic lesions upon soil transfer, normal development after juvenile stage.
*mlo17*	17	-	Mosaic	Chimeric with WT	Stunted growth. Normal development after juvenile stage.
*mlo3,4*	3	Bi	Homozygous		Slow growth, leaf senescence, non-flowering.
	4	Mono	Heterozygous		
*mlo3,13*	3	Bi	Homozygous		
	13	Mono	Mosaic		
*mlo13,17*	13	Mono	Mosaic		Black roots, loss of root gravitropism, narrow and thick leaves. Did not survive past 4th micropropagation.
	17	-	Mosaic	Chimeric with WT	
*mlo3,13,17*	3	Bi	Heterozygous		Slow growth and nonflowering.
	13	Bi	Mosaic		
	17	Mono	Mosaic	Chimeric with WT	
*qko #1*	3	Bi	Mosaic		
	4	-	Mosaic	Chimeric with WT	
	13	Bi	Mosaic		
	17	Bi	Mosaic		
*qko #2*	3	Bi	Homozygous		Stunted growth, premature senescence, did not survive.
	4	Mono	Heterozygous		
	13	Mono	Heterozygous		
	17	-	Mosaic	Chimeric with WT	

Type of editing: Mono/Biallelic refers to one or both alleles being edited. Mutation type: Homozygous refers to the same mutation in both alleles; heterozygous refers to different mutations in the alleles; mosaic refers to more than two types of editing; chimeric refers to the presence of unedited wild-type (WT) sequences alongside edited sequences. -: refers to a level of editing that was below a biallelic or monoallelic expected percentage.

## Data Availability

The datasets supporting the conclusions of this article are included within the article and its Supplementary Materials.

## Supplementary Materials

The following supporting information can be downloaded at: https://www.mdpi.com/article/10.3390/plants15203168/s1, Figure S1: Schematic illustration of the Single Transcript Unit transgenic cassette used for the expression of the CRISPR-Cas9 gene with the sgRNA array; Figure S2: Specificity of sgRNAs across the MLO gene targets. Each sgRNA was tested for cleavage activity on all four MLOs in an in vitro cleavage assay. Respective full-length MLO genes cloned into the pGMT vector were used as template DNA along with specific sgRNAs and Cas9 protein in the in vitro cleavage reaction. Target MLO gene-containing plasmids are linearized (~5kb), while the non-target MLO plasmids show nicked relaxed (slower migration), and supercoiled forms (faster migration) of the circular plasmids; Figure S3: Comparative analysis of the editing performance deduced from DECODR analysis and long-read sequence technology; Figure S4: Genotyping Analysis of the four mlo genes on qko #1 mutant using long-read sequencing. Alignment on the target sequence (sgRNA sequence + PAM). For each identified sequence, the percentage represents the number of reads relative to the total number of reads, accounting for sequencing errors; Figure S5: Stereoscopic observation of germination and growth of *E. necator* spores on infected leaves of WT microvine leaves and of *mlo* mutants 24 hpi; Figure S6: Wild-type microvine plant and *mlo* mutants growing in the greenhouse. Plants show 7 months of growth after spur-pruning in March 2026. The older bottom leaves showing natural senescence were not trimmed; Table S1: MLO gene nomenclature used in this study and cross-referenced with alternative names used in other studies; Table S2: Timeline of the critical infection stages of *Erysiphe necator* in grapevine leaves; Table S3: Single guide RNA sequences. The CRISTA score ranges from 0 to 1, where values closer to 1 indicate a higher predicted likelihood that Cas9 will cut that DNA sequence; Table S4: Overall performance of CRISPR-mediated editing based on the number of edited transgenic lines; Table S5: The overall editing for the four MLO genes was calculated for each *mlo* gene in the mutants; Table S6: Genotyping, sequencing and real-time PCR primers used for analysis of MLO mutants.
