# Supplementary material for: CRISPR-Cas9 Editing of Grapevine Clade V-Related MLO Genes: Grapevine Powdery Mildew Resistance (Erysiphe necator) and Plant Development Effects
Source: Plants (Basel). 2026 Oct 16;15(20):3168. doi: 10.3390/plants15203168 (PMC13708939; doi:10.3390/plants15203168)
Supplement: Supplementary file 1 [file plants-15-03168-s001.zip › plants-4591139-supplementary.pdf]

Supplementary Materials

CRISPR-Cas9 editing of grapevine clade V-related *mlo* genes revealed new insights into the contribution of each *mlo* gene to grapevine powdery mildew resistance (*Erysiphe necator*) and plant development

Satyanarayana Gouthu and Laurent G. Deluc

Supplementary figures

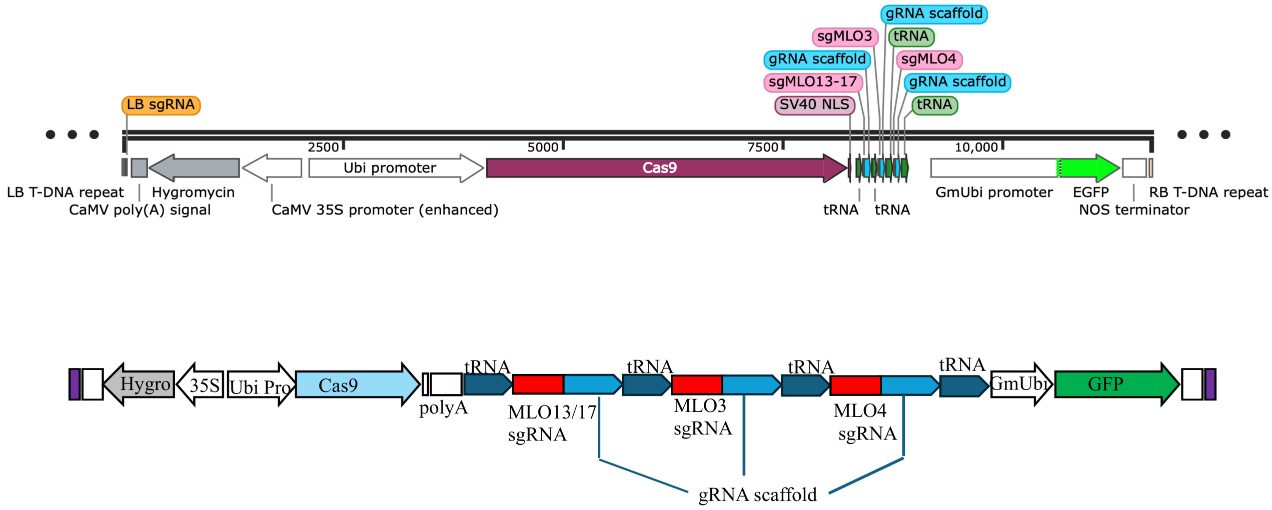

Figure S1. Schematic illustration of the Single Transcript Unit transgenic cassette used for the expression of the CRISPR-Cas9 gene with the sgRNA array.

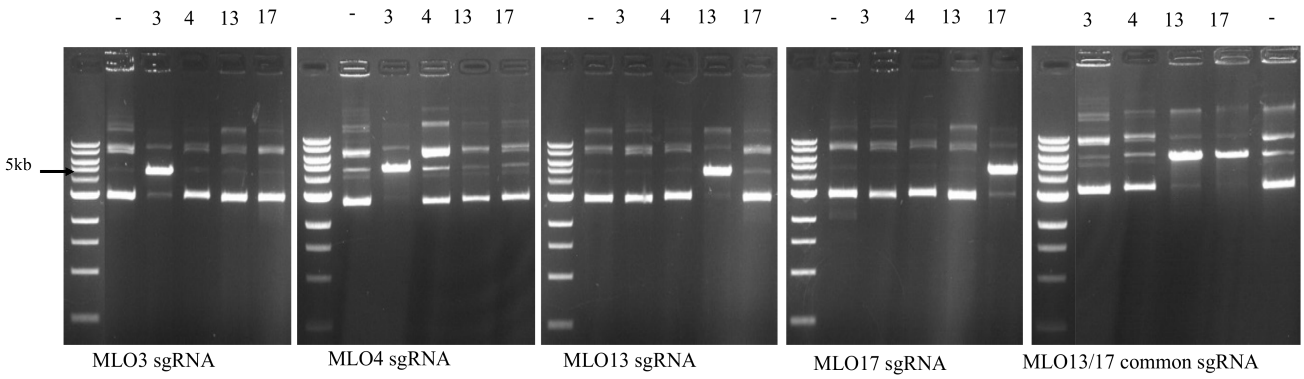

Figure S2. Specificity of sgRNAs across the MLO gene targets. Each sgRNA was tested for cleavage activity on all four MLOs in an in vitro cleavage assay. Respective full-length MLO genes cloned into the pGMT vector were used as template DNA along with specific sgRNAs and Cas9 protein in the in vitro cleavage reaction. Target MLO gene-containing plasmids are linearized (~5kb), while the non-target MLO plasmids show nicked relaxed (slower migration), and supercoiled forms (faster migration) of the circular plasmids.

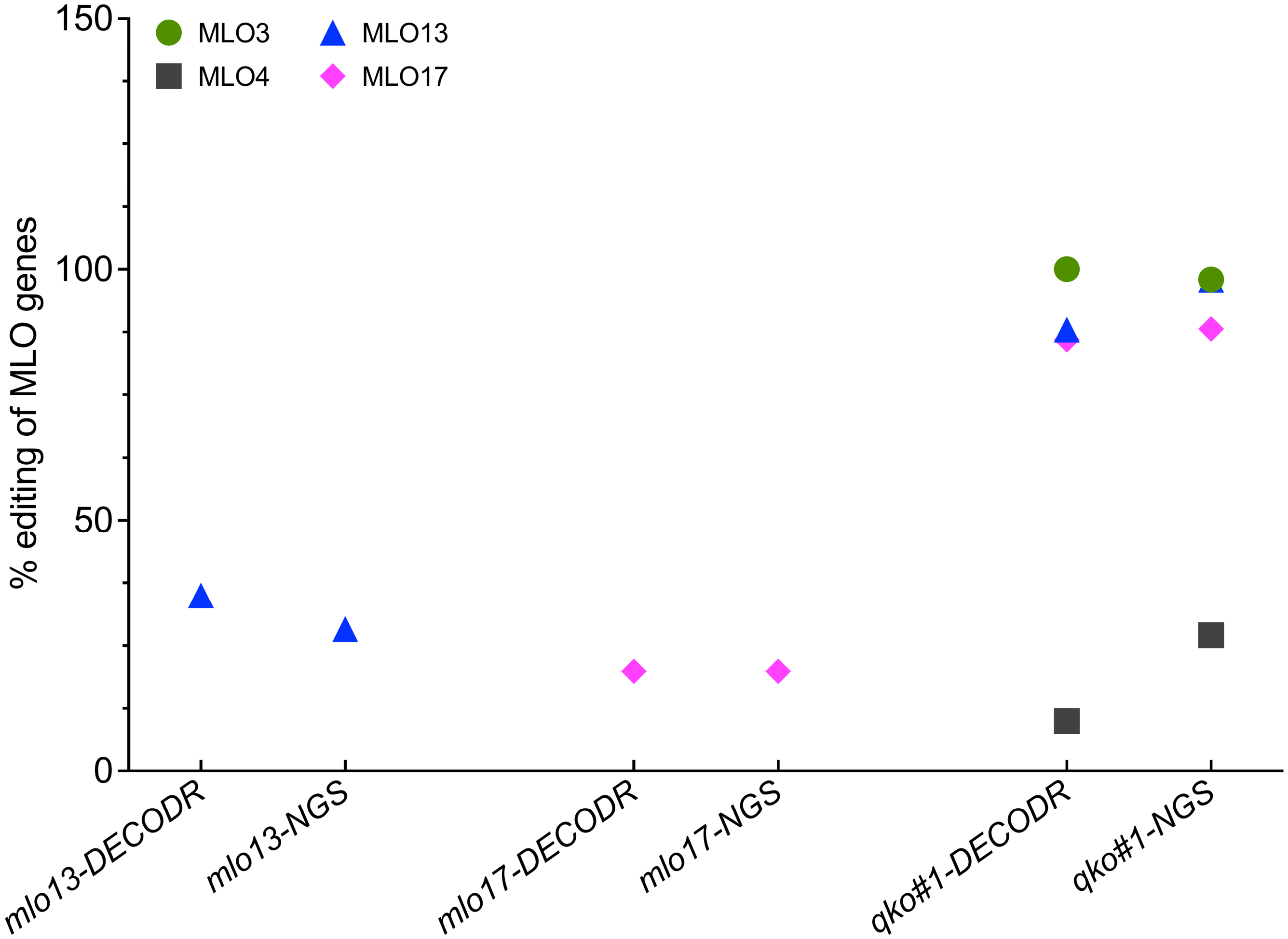

Figure S3. Comparative analysis of the editing performance deduced from DECODR analysis and long-read sequence technology.

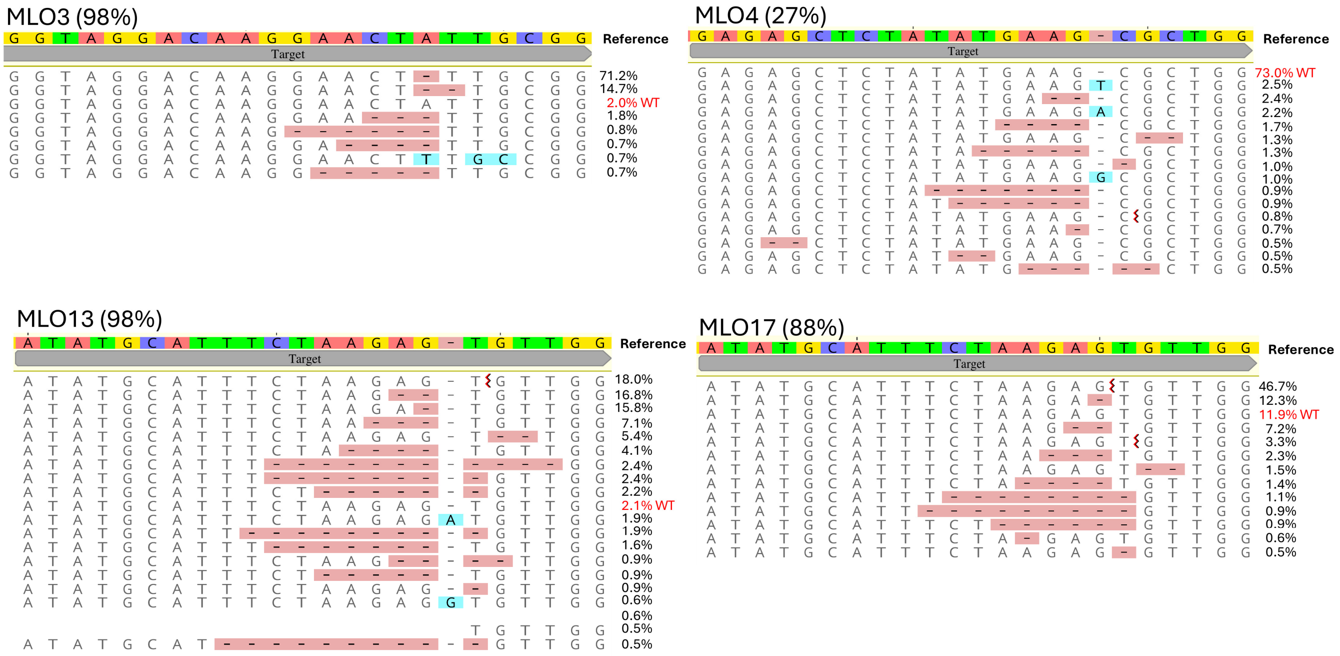

Figure S4. Genotyping Analysis of the four mlo genes on qko#1 mutant using long-read sequencing. Alignment on the target sequence (sgRNA sequence + PAM). For each identified sequence, the percentage represents the number of reads relative to the total number of reads, accounting for sequencing errors.

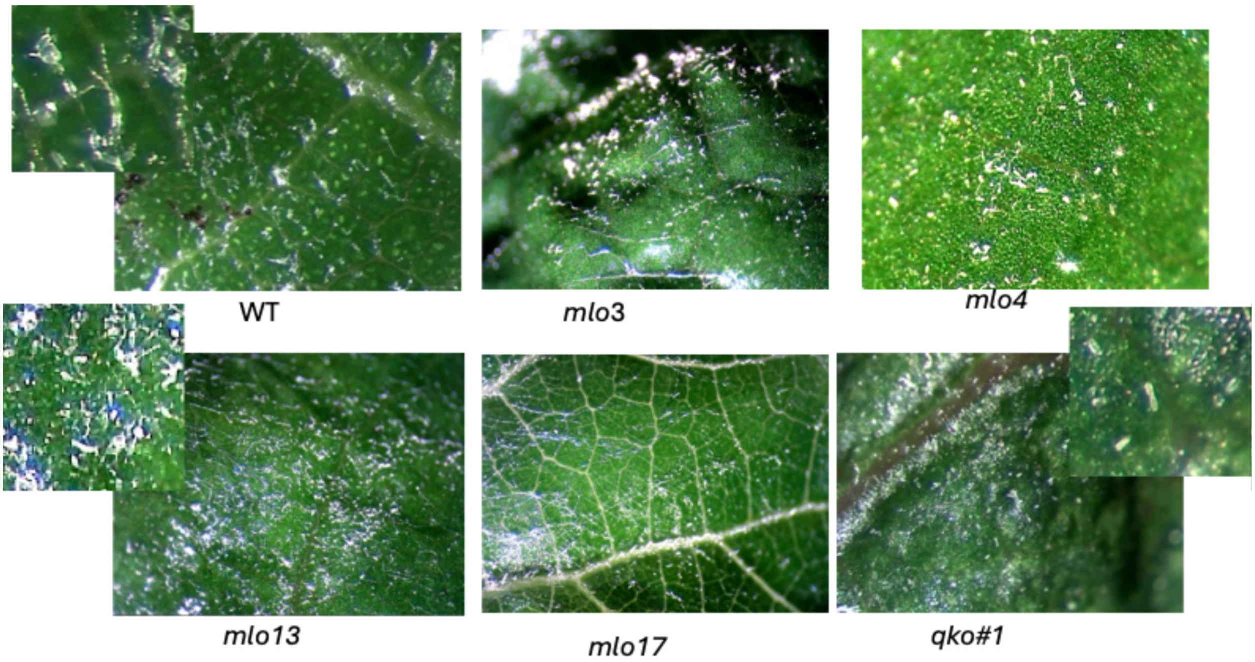

Figure S5. Stereoscopic observation of germination and growth of *E. necator* spores on infected leaves of WT microvine leaves and of *mlo* mutants 24hpi.

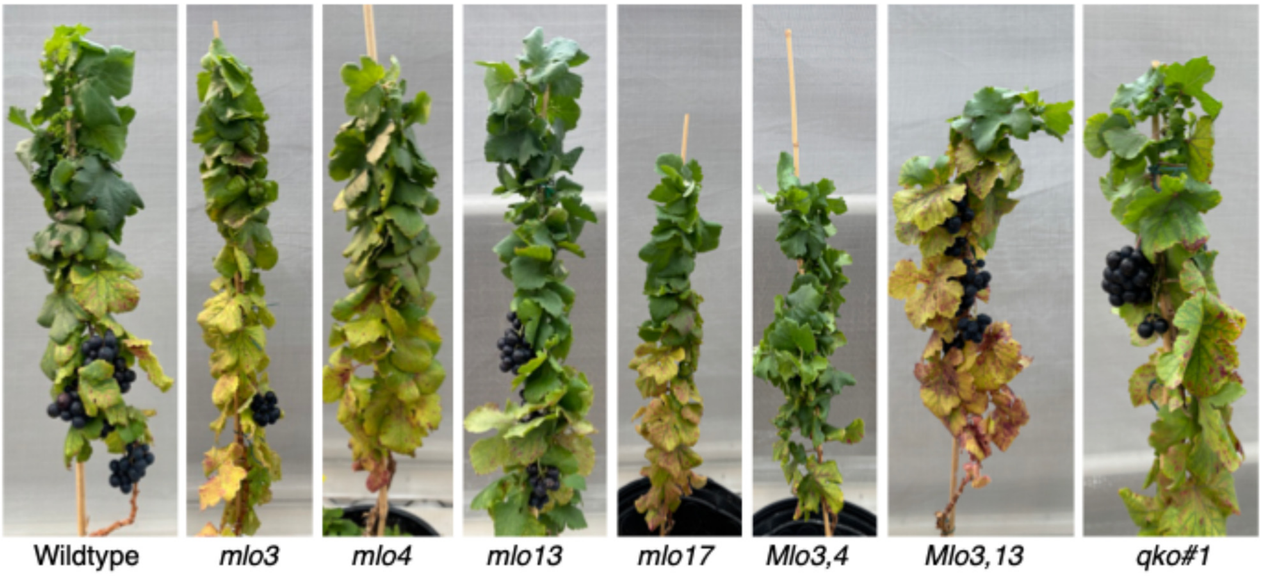

Figure S6. Wild-type microvine plant and *mlo* mutants growing in the greenhouse. Plants show 7 months of growth after spur-pruning in March 2026. The older bottom leaves showing natural senescence were not trimmed.

Supplementary tables

Table S1. MLO gene nomenclature used in this study and cross-referenced with alternative names used in other studies.

Gene name[1]	Alternative name[2]	V3-COST Annotation[3]
VvMLO3	*VvMLO11*	Vitvi08g01055
VvMLO4	*VvMLO13*	Vitvi06g00308
VvMLO13	*VvMLO6*	Vitvi13g00579
VvMLO17	*VvMLO7*	Vitvi13g00578

1 Feechan et al. [1], followed in the current study; 2 Winterhagen et al. [22] and Pessina et al. [4]; 3 Canaguier et al. [24]. Genome-sequence resource supplied in the original: http://urgi.versailles.inra.fr/Species/Vitis/Data-Sequences/Genome-sequences.

Table S2. Timeline of the critical infection stages of Erysiphe necator in grapevine leaves.

Stages	Earliest time post-inoculation	Approximate time length	Ref.
Conidial adhesion	Minutes following contact	Conidia rapidly adhere on the leaf surface through hydrophobic interactions and extracellular adhesive material	[29]
Conidial germination	2 hpi	Germination begins within hours under favorable humidity/temperature	[29]
Appressorium and penetration	6 hpi	Histological observations by 24–48 hpi have appressoria/haustoria visible	[30]
Haustorium formation	24 hpi	Gene expression atlas identifies haustorial structures by 24 hpi	[31]
Hyphal growth and colony expansion	48 hpi	Progressive epiphytic mycelium visible by 2–3 dpi in controlled inoculations	[29]
Sporulation/latent period	5 dpi	New conidia produced 5–10 dpi under optimal conditions	[29]

Table S3. Single guide RNA sequences. The CRISTA score ranges from 0 to 1, where values closer to 1 indicate a higher predicted likelihood that Cas9 will cut that DNA sequence.

Target gene	sgRNA	PAM	strand	Position from ATG	Exon #	CRISTA score [44]
MLO3	GGTAGGACAAGGAACTATTG	CGG	+	411-430	3rd	0.869
MLO4	GAGAGCTCTATATGAAGCGC	TGG	+	239-258	2nd	0.934
MLO13	GAAATGCATATCTTTGAAAT	GGG	-	815-795	3rd	0.900
MLO17	CACTTGGCACCCTTGTAAAA	AGG	+	1114-1133	3rd	0.903
Common MLO13/17	ATATGCATTTCTAAGAGTGT	TGG	+	805-824	3rd	0.907

Table S4. Overall performance of CRISPR-mediated editing based on the number of edited transgenic lines.

Gene	Number of transgenic lines screened	Number of transgenic lines with target edited	Efficiency (%)
MLO3	61	19	31
MLO4	65	8	12
MLO13	57	13	22
MLO17	64	11	17

Table S5. The overall editing for the four MLO genes was calculated for each mlo gene in the mutants.

Mutant name	Gene	Indel	Editing (%)	Frameshift mutation (%)	R^2
mlo3	*MLO3*	-1	100	100	1.00
mlo4	*MLO4*	+1	80	80	0.99
mlo13	*MLO13*	+1, -3, -84	35, 9, 8	35	0.62
mlo17	*MLO17*	-1, -4, -3	15, 10, 8	25	0.97
mlo3,4	*MLO3*	-2	100	100	1.00
	MLO4	+1	41	41	0.98
mlo3,13	*MLO3*	-1	100	100	0.96
	MLO13	+1, -1, -3	36, 11, 9	47	0.81
mlo13,17	*MLO13*	+1, -1, -3	36, 12, 8	48	0.72
	MLO17	-2	8	8	0.95
mlo3,13,17	*MLO3*	-1, -2	58, 31	89	0.98
	MLO13	-1, -2, +1	38, 27, 26	92	0.93
	MLO17	+1, -1, -2	12, 14, 22	48	0.96
qko #1	*MLO3*	-1	94	94	0.97
	MLO4	+2	25	25	0.98
	MLO13	+2, +1, -1	16, 13, 12	41	0.97
	MLO17	-1, -2	18, 8	26	0.98
qko #2	*MLO3*	-1, -2	18, 82	90	0.98
	MLO4	+1	51	51	0.99
	MLO13	0, -1, -2, -3	59, 16,13, 12	40	0.83
	MLO17	0, -1, -2	74, 18, 8	26	0.98

Table S6. Genotyping, sequencing and real-time PCR primers used for analysis of MLO mutants.

(A) Genotyping primers

Mutant	Forward primer (5′-3′)	Reverse primer (5′-3′)	Nested primer
MLO3	TAAGGGATCAAAGGATCGA	CCTTCTCTTGCCATAATCT	ACGAGCTCTTTATGAAGCAC
MLO4	GGCAACCGGAGGAAGGTCG	TCTCTTCTTCAGACTTGCTGC	GTACATCATTCATCTCACTG
MLO13	TGGTTAGCAAGAAGAAACAA	GAGCTGATGGATCCCATAG	TGGCTTCTTCTCATTGAGA
MLO17	TGGTTAAAAGGCAGACACAGG	GAGTTGATCAATCCCATAGG	TCATTGAGAGTAGTTCTTCC

(B) Real-time PCR primers

Gene	Forward primer (5′-3′)	Reverse primer (5′-3′)
MLO3	CAGCCCATGGACTTTTCATTCG	TTCGTTGCCCACAGCTATAC
MLO4	AGCCAACGATCTTCCATGAG	GGCCATCTTGACATGGGTGT
MLO13	GAAAGCCTCTCTCTCTCTATTTCC	TTTCTGCTGGTGCTCAGCTCTC
MLO17	AGGGACAAGGGATTGGAGAC	AAATACAAGCCGCAGAAAGG
ACTIN1	GTGTGGAGGGATTTATCTGTAATG	CAATCACTCTCCTGCTACAAAC
EnUNC	CCGCCAGAGACCTCATCCAA	TGTGCGTTCAAACATTCGATGA

References are numbered consistently with the main manuscript.

References

1. Feechan, A.; Jermakow, A.M.; Torregrosa, L.; Panstruga, R.; Dry, I.B. Identification of grapevine MLO gene candidates involved in susceptibility to powdery mildew. *Funct. Plant Biol.* **2008**, *35*, 1255–1266. https://doi.org/10.1071/fp08173.

4. Pessina, S.; Lenzi, L.; Perazzolli, M.; Campa, M.; Dalla Costa, L.; Urso, S.; Valè, G.; Salamini, F.; Velasco, R.; Malnoy, M. Knockdown of MLO genes reduces susceptibility to powdery mildew in grapevine. *Hortic. Res.* **2016**, *3*, 16016. https://doi.org/10.1038/hortres.2016.16.

22. Winterhagen, P.; Howard, S.F.; Qiu, W.; Kovács, L.G. Transcriptional Up-Regulation of Grapevine MLO Genes in Response to Powdery Mildew Infection. *Am. J. Enol. Vitic.* **2008**, *59*, 159–168.

24. Canaguier, A.; Grimplet, J.; Di Gaspero, G.; Scalabrin, S.; Duchêne, E.; Choisne, N.; Mohellibi, N.; Guichard, C.; Rombauts, S.; Le Clainche, I.; Bérard, A.; Chauveau, A.; Bounon, R.; Rustenholz, C.; Morgante, M.; Le Paslier, M.-C.; Brunel, D.; Adam-Blondon, A.-F. A new version of the grapevine reference genome assembly (12X.v2) and of its annotation (VCost.v3). *Genom. Data* **2017**, *14*, 56–62. https://doi.org/10.1016/j.gdata.2017.09.002.

29. Gadoury, D.M.; Cadle-Davidson, L.; Wilcox, W.F.; Dry, I.B.; Seem, R.C.; Milgroom, M.G. Grapevine powdery mildew (Erysiphe necator): a fascinating system for the study of the biology, ecology and epidemiology of an obligate biotroph. *Mol. Plant Pathol.* **2012**, *13*, 1–16. https://doi.org/10.1111/j.1364-3703.2011.00728.x.

30. Feechan, A.; Kabbara, S.; Dry, I.B. Mechanisms of powdery mildew resistance in the Vitaceae family. *Mol. Plant Pathol.* **2011**, *12*, 263–274. https://doi.org/10.1111/j.1364-3703.2010.00668.x.

31. Bonarota, M.-S.; Garcia, J.F.; Massonnet, M.; Zaccheo, M.; Figueroa-Balderas, R.; Cochetel, N.; Cantu, D. Dual Single-Nucleus Gene Expression Atlas of Grapevine and Erysiphe necator During Early Powdery Mildew Infection. *Mol. Plant-Microbe Interact.* **2025**, *38*, 1016–1030. https://doi.org/10.1094/mpmi-08-25-0099-r.

44. Abadi, S.; Yan, W.X.; Amar, D.; Mayrose, I. A machine learning approach for predicting CRISPR-Cas9 cleavage efficiencies and patterns underlying its mechanism of action. *PLoS Comput. Biol.* **2017**, *13*, e1005807. https://doi.org/10.1371/journal.pcbi.1005807.
